# A novel role for signal transducer and activator of transcription 5b (STAT5b) in β_1_-integrin-mediated human breast cancer cell migration

**DOI:** 10.1186/bcr2341

**Published:** 2009-07-24

**Authors:** Teresa M Bernaciak, Jessica Zareno, J Thomas Parsons, Corinne M Silva

**Affiliations:** 1 Department of Microbiology, University of Virginia, 1300 Jefferson Park Avenue, Charlottesville, VA 22908, USA; 2 Department of Medicine, University of Virginia, 1215 Lee Street, Charlottesville, VA 22908, USA; 3 Department of Cell Biology, University of Virginia, 1300 Jefferson Park Avenue, Charlottesville, VA 22908, USA; 4 Cancer Center, University of Virginia, 1222 Jefferson Park Avenue, Charlottesville, VA 22908, USA

## Abstract

**Introduction:**

Signal transducer and activator of transcription (STAT) 5b is a transcription factor involved in pro-proliferative and pro-survival signaling in a number of solid tumors, including breast cancer. The contribution of STAT5b to breast cancer cell motility has not been explored. This work aims to elucidate the role of STAT5b in breast cancer cell migration.

**Methods:**

STAT5b was knocked down by using siRNA in two aggressive, highly migratory breast cancer cell lines (BT-549 and MDA-MB-231), and transwell migration assays were performed to determine the importance of STAT5b for their migration. Knockdown-rescue experiments were used to validate the specificity of STAT5b knockdown and to determine which regions/functions of STAT5b are necessary for its role in migration. Live-cell imaging of wound healing and spreading was carried out to examine cell morphology and motility after STAT5b knockdown.

**Results:**

Knockdown of STAT5b, but not STAT5a, inhibited migration of BT-549 and MDA-MB-231 breast cancer cells to serum by 60% to 80%, and inhibited migration equally over a range of serum concentrations (0.1% to 10% serum). Migratory inhibition upon STAT5b knockdown could be rescued by reintroduction of wild-type STAT5b, as well as Y699F- and dominant-negative STAT5b mutants, but not an SH2 domain defective R618K-STAT5b mutant. β_1_- integrin-mediated migration of breast cancer cells to fibronectin was inhibited with STAT5b knockdown, and loss of STAT5b correlated with loss of directional migration and formation of multiple, highly contractile protrusions upon attachment to fibronectin.

**Conclusions:**

The data presented here demonstrate that STAT5b is integral to breast cancer cell migration and identify a novel, SH2-dependent function of STAT5b in regulating β_1_-integrin-mediated migration of highly aggressive breast cancer cells.

## Introduction

Breast cancer is the second most common cancer in American women. Despite improvements in detection and the development of new treatment strategies, the American Cancer Society estimates that more than 180,000 new cases of breast cancer will be diagnosed, and more than 40,000 women will die of breast cancer this year alone. Because many cancers arise from dysregulation of signaling pathways found in normal cells, one of the difficulties in treating cancers is identifying cancer-specific therapeutic targets. Current targeted therapies have not been as successful as anticipated. This lack of success is due in part to the ability of cancer cells to upregulate alternative signaling pathways to promote growth and tumor progression. Many tumorigenic signaling pathways converge on common nuclear transcription factors, and therefore, targeting these downstream proteins may be more effective [1].

One such group of transcription factors is the signal transducer and activator of transcription (STAT) family. STATs are a family of transcription factors activated by cytokines or growth factors or both. Seven members of the STAT family are known: STAT 1, 2, 3, 4, 5a, 5b, and 6. STAT proteins are latent in the cytoplasm and require phosphorylation of a conserved C-terminal tyrosine residue for activation. This allows dimerization to occur between the phosphorylated tyrosine of one STAT and the Src homology 2 (SH2) domain of another. Active dimers are translocated to the nucleus, where they bind DNA and regulate gene transcription. STAT proteins regulate transcription of genes involved in a variety of biologic processes, including proliferation, survival, and angiogenesis, all of which are involved in cancer development and progression. Thus, it is not surprising that in the last several years, a role for STATs in tumorigenesis has emerged. Activation of STAT5a and STAT5b occurs in a variety of cancers including both hematopoietic cancers and solid tumors, such as those of the breast, prostate, lung, head and neck, and brain [2,3]. STAT5a and STAT5b regulate the transcription of the pro-proliferative genes *c-myc *and *cyclin D1 *and the anti-apoptotic genes *Bcl-xL *and *Pim-1*, to stimulate tumor growth and survival [4-8]. In addition, STAT5b has been implicated in prostate cancer cell invasion [9].

To date, most of the work examining STAT5b in breast cancer has focused on its pro-proliferative function, and its role in breast cancer cell migration has not been examined. Importantly, a recent study investigating the effects of STAT5a on breast cancer cell migration and invasion showed that prolactin (Prl)-induced activation of STAT5a inhibited migration and invasion of BT-20 and T-47D human breast cancer cells [10]. STAT5a and STAT5b, although highly homologous, are encoded by two separate genes and function independently in mammary gland development. STAT5a is necessary for lobuloalveolar outgrowth and lactation mediated by Prl signaling, whereas STAT5b is vital for establishing growth hormone (GH)-directed sexual dimorphism [11,12].

Given this background, we sought to investigate the potential role of STAT5b, specifically, in the migration of two highly aggressive, highly migratory breast cancer cell lines. We found that STAT5b knockdown inhibited serum- and fibronectin-stimulated migration of both BT-549 and MDA-MB-231 human breast cancer cell lines in a transwell assay. This inhibition was rescued by co-expression of wild-type, Y699F-, and dominant-negative STAT5b but not STAT5b containing a mutation in the SH2 domain. With real-time imaging, we showed that knockdown of STAT5b resulted in decreased directional migration and the formation of multiple protrusions, giving rise to an overall reduction in motility. These results establish, for the first time, an important SH2-dependent function of STAT5b in aggressive breast cancer, further defining its role in tumorigenesis and supporting its potential as a therapeutic target for the treatment of breast cancer.

## Materials and methods

### Cell culture

BT-549 and MDA-MB-231 human breast cancer cell lines were obtained from the American Type Culture Collection (Manassas, VA). Cells were passaged twice per week and maintained in Dulbecco's Modified Eagle Medium (DMEM) supplemented with 10% fetal bovine serum (FBS). All tissue-culture reagents were purchased from Invitrogen (Gaithersburg, MD).

### siRNA Transfection

BT-549 and MDA-MB-231 cells were transfected with siGENOME SMARTpool siRNA targeting human STAT5b or individual custom oligonucleotides specific for STAT5a or STAT5b (siGENOME STAT5b SMARTpool duplex #3), or luciferase duplex control, all purchased from Dharmacon (Lafayette, CO). Transfections were performed by using either Oligofectamine (Invitrogen) or Amaxa nucleofection (Amaxa/Lonza, Walkersville, MD), by using solution T and program X-013 (MDA-MB-231 cells) or A-023 (BT-549 cells) as per manufacturers' instructions. For knockdown-rescue experiments, cells were transfected simultaneously with siSTAT5b SMARTpool duplex #3 and HA-tagged wild-type-, Y699F-, dominant-negative-, or R618K-STAT5b engineered to be immune to knockdown by introduction of four silent point mutations in the siRNA target sequence. These point mutations were introduced by using QuikChange site-directed mutagenesis (Stratagene, La Jolla, CA), and constructs were sequenced to verify mutations.

### Immunoblotting

Cells were lysed in RIPA buffer (150 mmol/L NaCl, 50 mmol/L Tris, pH 7.4, 1% deoxycholate, 1% Triton X-100, 5 mmol/L EDTA) containing protease inhibitor cocktail (Calbiochem, San Diego, CA) and sodium orthovanadate (Sigma, St. Louis, MO), and boiled in 2× Laemmli buffer containing β-mercaptoethanol or 20 mmol/L dithiothreitol for 5 minutes at 100°C. Protein lysates were separated on 7.5% or 12.5% polyacrylamide gels and transferred to nitrocellulose (Pall Corporation, Pensacola, FL). Membranes were blocked and incubated with primary antibodies in TBST (150 mmol/L NaCl, 0.1% Tween 20, 50 mmol/L Tris, pH 8.0) containing 5% nonfat dry milk or 3% BSA. STAT5a- and STAT5b-specific polyclonal antibodies were developed by our laboratory, as previously described [13]. Monoclonal anti-β-actin antibody was purchased from Santa Cruz Biotechnology (Santa Cruz, CA). HA monoclonal antibody was obtained from the University of Virginia hybridoma facility. Secondary antibodies were applied in TBST and were HRP-conjugated sheep anti-mouse or donkey anti-rabbit (GE Healthcare, Piscataway, NJ). The enhanced chemiluminescence detection kit (GE Healthcare) was used to detect antibody binding. Acrylamide was from Bio-Rad (Hercules, CA), prestained molecular weight standards were from Sigma, and all other reagents were of reagent or molecular biologic grade from Sigma.

### Transwell migration assays

BT-549 and MDA-MB-231 cells were transfected with siRNA, as described earlier. Seventy-two hours after transfection, 5 × 10^4 ^BT-549 cells or 1 × 10^5 ^MDA-MB-231 cells were plated in serum-free (DMEM/0.1% BSA) media into the upper chambers of BD BioCoat Control Chambers (BD Biosciences, San Jose, CA), and DMEM containing 0 to 10% FBS was placed in the lower chamber. For β_1_-integrin blocking experiments, cells were pretreated for 1 hour with DMSO (vehicle control) or 10 μg/ml monoclonal anti-human β_1_-integrin antibody (R&D Systems, Minneapolis, MN), and treatment was left on for the duration of the assay. For migration to extracellular matrix components, the undersides of filters were coated with 0 to10 μg/ml human plasma fibronectin (FN) (BD Biosciences) or recombinant human vitronectin (VN) (R&D Systems) overnight at 4°C, as indicated, and DMEM/0.1% BSA was used in both the upper and lower chambers. Plates were incubated at 37°C, and migration was allowed to proceed for 3 to 6 hours. After this time, nonmigratory cells in the upper chambers were removed with cotton swabs, and the remaining cells were stained with 0.1% crystal violet (Sigma) in 20% ethanol. Cells were counted by using a Zeiss Invertoskop light microscope. Four fields were counted on each of two filters. Results are expressed as average cells per field or relative migration (compared with control).

### Wound healing

Cell were plated on FN-coated (5 μg/ml) 35-mm Bioptechs delta T dishes (Fisher Scientific, Pittsburgh, PA). Confluent cell monolayers were wounded by using a 20-μl pipette tip, and cells migrating into the wound were filmed at 37°C with time-lapse microscopy by using a Nikon TE200 inverted microscope with a 20× differential interference contrast objective and a Bioptechs heated stage. Images were taken with a Hamamatsu Orca camera every 5 minutes for 6 hours and collected with Openlab software (Improvision, Lexington, MA). Movies were analyzed by using Image J Manual Tracking software (National Institutes of Health, Bethesda, MD). Single cell nuclei were tracked over time, and migratory speed was calculated by dividing the length of the migration path by the total movie time. Directional persistence was determined as the net displacement divided by the total length of the migration path.

### Total internal reflection fluorescence (TIRF) microscopy

Cells were transfected with siRNA oligonucleotides, mKO-paxillin, and GFP-speckle-actin by using Amaxa nucleofection, as described earlier. Seventy-two hours after transfection, cells were plated on FN (2 μg/ml), and after 20 to 30 minutes of spreading, TIRF images were acquired by using an inverted microscope, 60× objective (Olympus, model IX70). GFP was excited by using the 488-nm laser line of an Ar ion laser, and RFP was excited by using the 543-nm laser line of an He-Ne laser (Mells Griot, Carlsbad, CA). Time-lapse images were taken every 3 seconds for 5 minutes with a charge-coupled device camera (QImaging, Surrey, BC, Canada) and analyzed by using MetaMorph (MDS Analytical Technologies, Mississauga, Ontario, Canada). Protrusion rates were determined by using ImageJ Kymograph software (National Institutes of Health) and calculated as the length of the protrusion divided by the total time of the movie.

## Results

### STAT5b knockdown inhibits breast cancer cell migration

**Figure 1:**
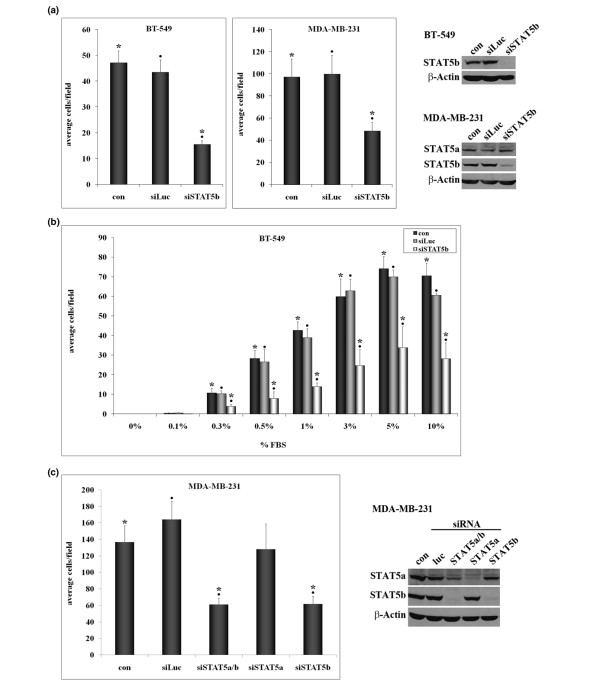
STAT5b knockdown inhibits breast cancer cell migration. **(a) **BT-549 or MDA-MB-231 breast cancer cells were transfected with no siRNA (con), control siRNA for luciferase (siLuc), siSTAT5b SMARTpool (BT-549 cells), or siSTAT5b SMARTpool duplex #3 (MDA-MB-231 cells). Seventy-two hours after transfection, cells were plated in serum-free media in trans-well chambers. Media containing 1% fetal bovine serum (FBS; BT-549) or 10% FBS (MDA-MB-231) were placed in the lower chambers. After 3 hours (BT-549) or 6 hours (MDA-MB-231), cells were fixed, stained with crystal violet, and the number of migratory cells was counted. Results are graphed as the average number of migratory cells per field ± SEM. One-way ANOVA with Tukey's post-test was used to determine statistical significance (*P *< 0.05) between the following: BT-549: con and siSTAT5b (*), siLuc and siSTAT5b (black circles); n = 5. MDA-MB-231: con and siSTAT5b (*), siLuc and siSTAT5b (black circles); n = 4. (b) BT-549 breast cancer cells were transfected and plated in serum-free media in trans-well chambers for 3 hours. Media containing varying concentrations of FBS (0.1% to 10%) were placed in the lower chambers. One-way ANOVA with Tukey's post-test was used to determine statistical significance (*P *< 0.05) between the following: con and siSTAT5b (*), siLuc and siSTAT5b (black circles); n = 3. **(c) **MDA-MB-231 cells were transfected with no siRNA (con), control siRNA for luciferase (siLuc), or siRNA specific to STAT5a/b (siSTAT5b siGENOME SMARTpool), STAT5a (siSTAT5a custom oligonucleotide), or STAT5b (siSTAT5b siGENOME SMARTpool oligonucleotide 3), and trans-well migration assays were performed as described. One-way ANOVA with Tukey's post-test was used to determine statistical significance (*P *< 0.05) between the following: con and siSTAT5a/b (*), con and siSTAT5b (*), siLuc and siSTAT5a/b (black circles), siLuc and siSTAT5b (black circles); n ≥ 5. (a and c) Whole-cell lysates from siRNA-transfected cells were immunoblotted with antibodies specific for STAT5a, STAT5b, and β-actin as a loading control.

To determine the importance of STAT5b for breast cancer cell migration, we used siRNA transfection to knock down STAT5b in the highly migratory BT-549 breast cancer cell line. Knockdown of STAT5b to virtually undetectable levels in these cells inhibited their migration to serum by approximately 70% (Figure 1A). To ensure that this effect was not unique to one cell line, we tested the effect of STAT5b siRNA knockdown on migration of MDA-MB-231 cells, another highly aggressive, migratory breast cancer cell line. In these cells, knockdown of STAT5b also inhibited migration to serum, by approximately 50% (Figure 1A).

It is well established that STAT5b promotes cell-cycle progression and survival of breast cancer cells [14-19]. To determine whether knockdown of STAT5b affected proliferation or survival under the conditions of our migration experiments, we performed trypan blue assays on knockdown cells 72 hours after transfection. In both BT-549 and MDA-MB-231 cells, knockdown of STAT5b did not significantly alter the total number of adherent cells or the viability of adherent cells over a 6-hour period, which is the maximum length of our transwell migration assays (data not shown). Thus, we conclude that the inhibition of migration after knockdown of STAT5b is not a result of secondary effects on adherence, viability, or survival.

By using BT-549 cells, we determined the optimal migration conditions for trans-well assays to be the migration to 1% serum over a 3-hour period. Because these cells require a low dose of serum to migrate, we used them to determine whether increasing concentrations of serum could overcome the effect of STAT5b knockdown. As seen in Figure 1B, migration to serum was dose dependent, with 3% to 10% serum being optimal. Knockdown of STAT5b significantly inhibited migration of BT-549 cells by 60% to 80% at all serum concentrations. Similar results were obtained in the MDA-MB-231 cells (data not shown). Thus, increasing the concentration of serum components did not overcome the effect of STAT5b knockdown on inhibiting migration. However, migration was not completely abrogated after knockdown of STAT5b in either cell line. The cells retained their capacity to migrate at a similar "basal" level across all serum concentrations tested. This indicates that both STAT5b-dependent and STAT5b-independent pathways may be responsible for migration of these cells to serum.

Because STAT5a has been implicated in breast cancer migration [10], we investigated the role of STAT5a on migration in our model system. We chose to use the MDA-MB-231 breast cancer cell line because they are highly migratory and express both STAT5a and STAT5b proteins, whereas BT-549 breast cancer cells express only STAT5b (data not shown). To target STAT5b specifically in the MDA-MB-231 cells, we used one of the individual oligonucleotides from the SMARTpool, which had no effect on STAT5a levels (Figure 1C, lane *5*). The total SMARTpool, previously used in the BT-549 cells, was used to determine the effect of dual knockdown of STAT5a and STAT5b in our MDA-MB-231 model system. Whereas knockdown of STAT5b inhibited migration of MDA-MB-231 cells by greater than 50%, knockdown of STAT5a had no significant effect on migration of these cells (Figure 1C). Furthermore, knockdown of STAT5a in combination with STAT5b did not enhance the inhibition of migration due to knockdown of STAT5b alone. Therefore, STAT5b is necessary for optimal migration of highly aggressive breast cancer cells, whereas expression of STAT5a is not required and cannot compensate for loss of STAT5b.

**Figure 2:**
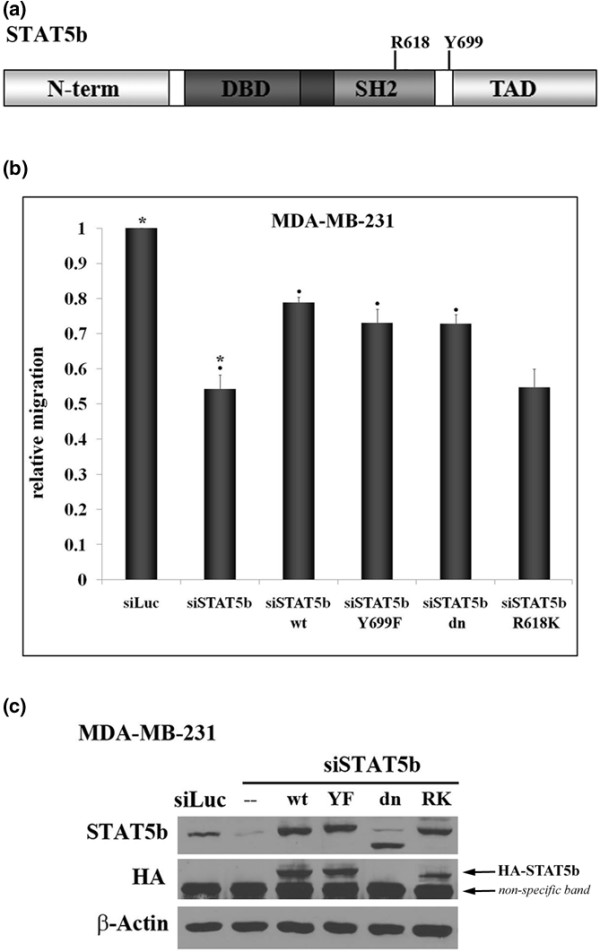
Expression of wild-type, Y699F-, or dominant-negative STAT5b rescues migration, but expression of R618K-STAT5b does not. **(a) **Domain structure of the STAT5b protein depicting the amino terminus (N-term), DNA-binding domain (DBD), Src homology 2 domain (SH2), transactivation domain (TAD), and the location of the conserved tyrosine residue, Y699, and arginine residue, R618. **(b) **MDA-MB-231 breast cancer cells were transfected with control siRNA to luciferase (siLuc) or siRNA specific to STAT5b (siSTAT5b siGENOME SMARTpool oligonucleotide #3) alone or in the presence of HA-tagged wild-type (wt-STAT5b), Y699F (YF-STAT5b), dominant-negative (dn-STAT5b), or R618K (RK-STAT5b) STAT5b constructs engineered to be immune to siRNA knockdown. Seventy-two hours after transfection, trans-well assays were performed for 6 hours, as described in Figure 1. Results are graphed as relative migration, compared with siLuc control. One-way ANOVA with Tukey's post-test was used to determine statistical significance (*p *< 0.05) between the following: siLuc and siSTAT5b (*), siSTAT5b and siSTAT5b + wt-STAT5b (black circles), siSTAT5b and siSTAT5b + YF-STAT5b (black circles), siSTAT5b and siSTAT5b + dn-STAT5b (black circles); n ≥ 4. Comparison of siSTAT5b and siSTAT5b + RK-STAT5b was not statistically significant. Whole-cell lysates from transfected cells were immunoblotted with antibodies specific for STAT5b, HA, or β-actin as a loading control.

To eliminate the possibility of off-target effects of STAT5b knockdown, we performed knockdown-rescue experiments. MDA-MB-231 cells were simultaneously transfected with STAT5b-specific siRNA and hemagglutinin (HA)-tagged wild-type STAT5b that is resistant to knockdown. Consistent with previous experiments, knockdown of STAT5b inhibited migration of MDA-MB-231 cells by approximately 60%. Re-introduction of wild-type STAT5b restored migration to approximately 76% of control levels (Figure 2B), confirming that the inhibition of migration with STAT5b knockdown is due to a direct effect of STAT5b on migratory pathways. In these experiments, transfection efficiency of siRNA was close to 100%, whereas transfection efficiency of rescue constructs was approximately 65% to 75% (data not shown).

### Expression of wild-type, Y699F-, or dominant-negative STAT5b rescues migration, but expression of R618K-STAT5b does not

Additional knockdown-rescue experiments were performed to identify the functional domains required for rescue of the siRNA phenotype. We introduced Y699F-, dominant-negative (dn)-, and R618K-STAT5b mutants to test the requirement of transcriptional activity and active Y699-SH2 STAT5b dimers for promoting migration. Y699F-STAT5b cannot be phosphorylated on the conserved tyrosine residue Y699, and dn-STAT5b lacks the C-terminal transactivation domain, rendering these constructs transcriptionally inactive [14,17,20]. R618K-STAT5b has a mutation in the conserved arginine required for SH2 domains to bind phosphorylated tyrosines, interfering with the ability of this mutant to form active dimers with Y699-phosphorylated STAT5b [21]. In migration assays, re-introduction of Y699F- and dn- STAT5b into MDA-MB-231 cells rescued migration to the same level as did rescue with wild-type STAT5b. In contrast, the R618K-STAT5b mutant could not rescue migration (Figure 2B). Taken together, these data indicate that STAT5b-mediated transcription is not necessary for regulating migration. Moreover, the SH2 domain has an integral role, separate from that of active dimer formation, in promoting migration.

### STAT5b knockdown inhibits β_1_-integrin-mediated migration to FN

We sought to determine the serum component responsible for migration of breast cancer cells as a means of gaining insight into those migratory signaling pathways to which STAT5b contributes. Despite previously published work, we did not observe migration of BT-549 or MDA-MB-231 cells to the chemokine stromal cell-derived factor-1β (SDF-1β) [22]. In addition, no migration was observed to lysophosphatidic acid (LPA), a major component of serum. Interestingly, in the absence of serum, neither cell line migrated to epidermal growth factor (EGF), despite expression of the epidermal growth factor receptor (EGFR). We showed previously that EGF stimulation of breast cancer cells leads to phosphorylation of STAT5b on Y699 and subsequent transcriptional activity [13,14,16,17]. Therefore, the lack of migration to EGF is consistent with the observation that Y699 phosphorylation and active transcription is not required for the STAT5b migratory function. Additionally, we did not detect either basal or serum-induced Y699 phosphorylation in these cells, further supporting the idea that STAT5b mediates migration to serum through a separate, transcription-independent pathway.

**Figure 3:**
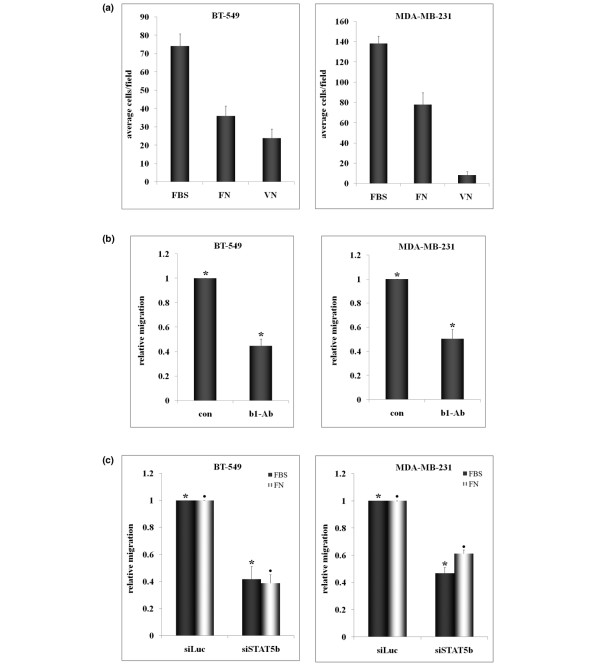
STAT5b knockdown inhibits β1-integrin-mediated migration to fibronectin (FN). **(a) **The undersides of trans-well filters were coated with 10 μg/ml FN or vitronectin (VN) overnight at 4°C. BT-549 and MDA-MB-231 cells were placed in serum-free media in upper chambers, and 1% fetal bovine serum (FBS) (BT-549) or 10% FBS (MDA-MB-231) medium was placed in the lower chambers for FBS controls. Serum-free medium was placed in lower chambers for FN and VN conditions. Migration was allowed to proceed for 3 hours (BT-549) or 6 hours (MDA-MB-231) (n = 3). (b) BT-549 and MDA-MB-231 cells were pretreated for 1 hour at 37°C with 10 μg/ml β_1_-integrin-blocking antibody or DMSO control (con). Cells were plated in trans-well chambers in the presence of blocking antibody, and migration to 1% FBS (BT-549) or 10% FBS (MDA-MB-231) was measured. Student's *t *test was used to determine statistical significance (*P *< 0.05) between the following: BT-549: con and antibody (*) (n = 6); MDA-MB-231: con and antibody (*) (n = 4). **(c) **The undersides of trans-well filters were coated with 3 μg/ml FN overnight at 4°C, and migration assays were performed with siRNA-transfected cells as described in part a. One-way ANOVA with Tukey's post-test was used to determine statistical significance (*P *< 0.05) between the following: BT-549: siLuc and siSTAT5b FBS (*), siLuc and siSTAT5b FN (black circles); n = 4. MDA-MB-231: siLuc and siSTAT5b FBS (*), siLuc and siSTAT5b FN (black circles); n = 3.

Next, we tested migration of cells to extracellular matrix components known to be present in serum, specifically fibronectin (FN) and vitronectin (VN). Both BT-549 and MDA-MB-231 cells migrated to FN, whereas only BT-549 cells migrated significantly to VN (Figure 3A). Therefore, to compare effects on both cell lines, we used fibronectin as the migratory stimulus. Cells attach to fibronectin and mediate signaling through integrin receptors comprising β_1_-integrin in combination with various α-integrins. To confirm that fibronectin was indeed a major serum component stimulating migration, we pretreated cells with a β_1_-integrin blocking antibody and measured migration to serum. Inhibition of β_1_-integrin receptor inhibited migration of both cell lines to serum by approximately 50% compared with the vehicle control (Figure 2B).

Nonspecific mouse IgG had no effect on migration (data not shown). Fibronectin dose curves were performed, and it was found that optimal migration of both BT-549 and MDA-MB-231 cell lines occurred at 2 to 3 μg/ml FN (data not shown). This is consistent with a study by Hayman and colleagues [23], which measured the level of FN in fetal bovine serum to be 25 μg/ml, or 2.5 μg/ml in 10% FBS/DMEM. To determine whether STAT5b is important in β_1_-integrin-mediated migration, we examined the effect of STAT5b knockdown on migration to FN. Knockdown of STAT5b inhibited migration of both cell lines to FN, to the same extent that knockdown inhibited migration to serum, approximately 40% to 60% (Figure 3C). Taken together, these results demonstrate that STAT5b is integral for β_1_-integrin-mediated migration of breast cancer cells to FN.

### STAT5b knockdown results in multiple protrusions and negatively affects directionality during wound closure

**Figure 4:**
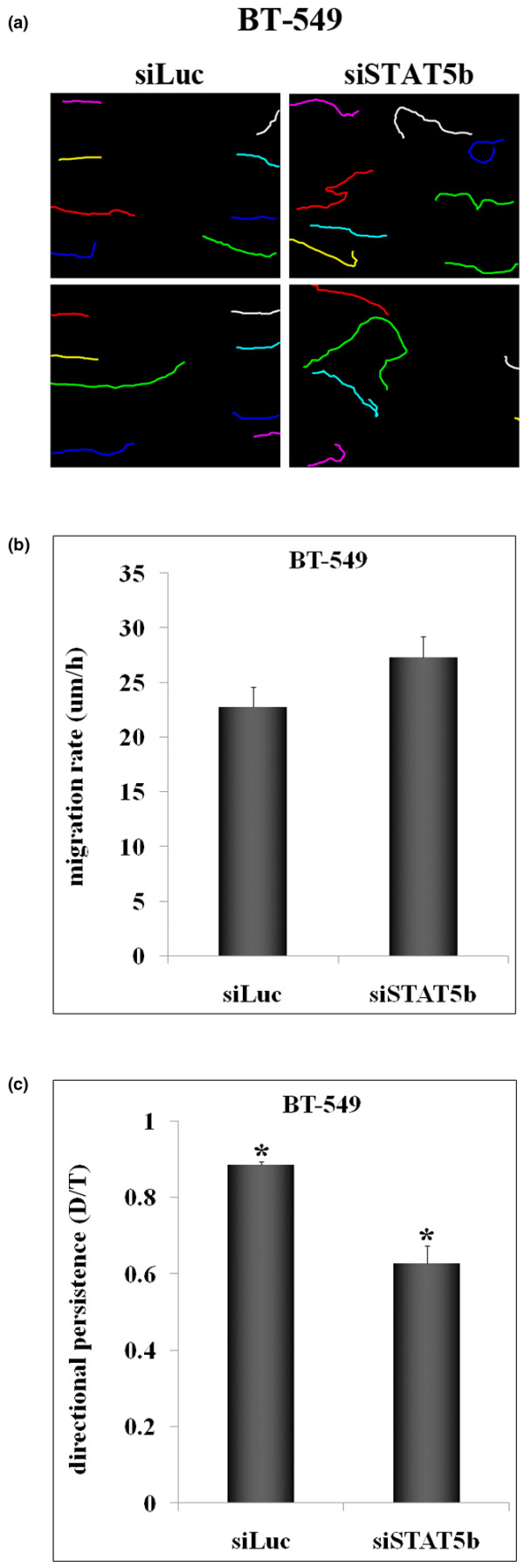
STAT5b knockdown negatively affects directionality during wound closure. Wounds were made in confluent monolayers of siRNA-transfected BT-549 cells, and time-lapse microscopy was used to track cell movement into the wound over a 6-hour period. **(a) **Progressive line images tracking single cell nuclei of cells along the wound edge. Two independent experiments are shown. **(b) **Migration rate (μm/h) was calculated by dividing the length of the migration path by the total movie time. **(c) **Directional persistence (D/T) was determined as the net displacement (D) divided by the total length of the migration path (T). (b, c) Values from at least four independent experiments were used for calculations (siLuc, n = 24; siSTAT5b, n = 32). Student's *t *test was used to determine statistical significance (*p *< 0.05) between siLuc and siSTAT5b (*). Comparisons of migration rate were not statistically significant.

Wound-healing assays were performed to examine cell morphology and motility during wound closure of cells plated on FN. After STAT5b knockdown, wounds were allowed to close for 6 hours, and time-lapse microscopy was used to track cell movement into the wound. Figure 4A depicts the paths of cells (tracked by single cell nuclei) along the wound edge. Control cells migrated in relatively straight lines into the wound area. In contrast, STAT5b-knockdown cells showed compromised directionality in movement, migrating both horizontally and vertically. To quantitate these results, we measured migration rate and directional persistence (Figure 4B and 4C). Knockdown of STAT5b had little effect on overall migration rate; however, cells deficient in STAT5b exhibited significant loss of directional persistence. Additionally, the number of cells with Golgi localized between the nucleus and leading edge during wound closure was significantly less after STAT5b knockdown (data not shown), suggestive of a defect in cell polarity. This is consistent with findings that STAT5b-knockdown cells migrate less efficiently in transwell assays (Figure 1).

**Figure 5:**
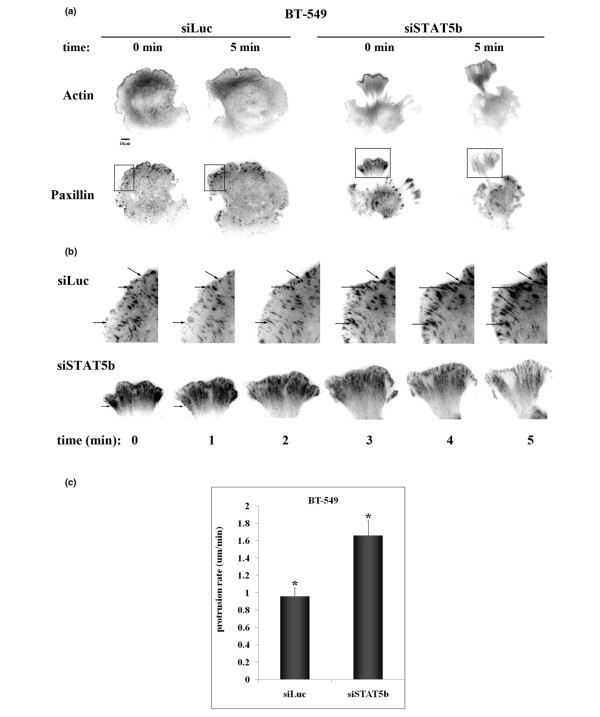
STAT5b-knockdown cells exhibit multiple protrusions with increased retraction. BT-549 cells were transfected with siRNA (siLuc or siSTAT5b), GFP-speckle-actin, and mKO-paxillin. Seventy-two hours after transfection, cells were plated on 3 μg/ml fibronection (FN) for 20 to 30 minutes, and TIRF time-lapse microscopy was performed. Images were taken every 3 seconds for 5 minutes at 60× magnification. (Line indicates 10 μm). **(a) **Still images representing actin and paxillin staining in control (siLuc) and STAT5b-knockdown (siSTAT5b) cells at start of filming (0 min) and end of filming (5 min). **(b) **Enlarged images depicting boxed areas from part a. Arrows identify single focal adhesions at each time point. **(c) **Protrusion rate (μm/min) was calculated as the length of the protrusion divided by the total time of the movie for at least three independent experiments (siLuc, n = 16; siSTAT5b, n = 13). Student's *t *test was used to determine statistical significance (*P *< 0.05) between siLuc and siSTAT5b (*).

To examine focal adhesion and actin dynamics at the membrane, total internal reflection fluorescence (TIRF) microscopy was used to image cells plated on FN. STAT5b-knockdown cells spread slightly faster on FN and exhibited a striking phenotype characterized by the formation of multiple irregular protrusions (Figure 5). Knockdown cells were highly contractile and displayed increased retrograde movement of cell adhesions. Analysis of focal adhesion turnover revealed that control cells underwent normal focal adhesion turnover, with small adhesions present at the leading edge, and mature, stable adhesions located farther inside the cell (Figure 5B). STAT5b-knockdown cells readily formed adhesions at the leading edge of protrusive lamellipodia, but over time, these protrusions contracted, and the adhesions underwent retrograde flow back into the cell (Figure 5B). As a whole, these data suggest that STAT5b contributes to the coordinated regulation of protrusive and contractive processes.

## Discussion

The work presented here establishes an important, previously undiscovered role for STAT5b in the migration of highly aggressive breast cancer cells. Knockdown of STAT5b inhibits migration of BT-549 and MDA-MB-231 human breast cancer cells to serum and fibronectin (Figures 1 and 3). This inhibition can be rescued by expression of wt-, Y699F-, or dn-STAT5b, but not with the SH2 domain mutant R618K-STAT5b (Figure 2). Upon attachment to fibronectin, STAT5b-knockdown cells form multiple, contractile protrusions resulting in loss of directionality and inefficient migration (Figures 4 and 5).

Knockdown of STAT5b inhibits migration of both BT-549 and MDA-MB-231 breast cancer cell lines to serum, whereas knockdown of STAT5a has no effect (Figure 1). These results may seem contradictory to those published by Sultan and colleagues [10], which reported a suppressive effect of STAT5a on breast cancer cell migration. However, in those studies, STAT5a was overexpressed in the moderately migratory BT-20 and T-47D breast cancer cell lines, which contain little to no endogenous STAT5a. The STAT5a and STAT5b expression pattern in those cell lines is very different from that seen in MDA-MB-231 cells, and consequently, the signaling may differ. Moreover, the role of endogenous STAT5b was not investigated in the studies of Sultan and associates. We and others found that STAT5b is the predominant STAT5a/b protein expressed in breast cancer cell lines and tissues, and that STAT5b, not STAT5a, mediates proliferation of breast cancer cells [10,14,16]. Differential expression or activity (or both) of STAT5a and STAT5b has been reported in other cancer model systems [9,24,25]. STAT5b levels and phosphorylation are elevated in SCCHN tumors compared with control mucosa, whereas STAT5a levels to do not change [24]. Consistent with these findings, antisense inhibition of STAT5b, but not STAT5a, inhibits *in vivo *growth of SCCHN xenografts [24]. In prostate cancer, differential STAT5a and STAT5b protein expression can be correlated with metastatic potential. STAT5a is expressed in nonmetastatic C1D mouse prostate cancer cells, but not in their metastatic C2H counterparts, whereas STAT5b is expressed in both [9]. Additionally, STAT5a is expressed in LNCaP human prostate cancer cells but not the more highly migratory PC-3 prostate cancer cell line, but STAT5b levels are comparable [9]. For these reasons, it is imperative to determine the individual contributions of STAT5a and STAT5b, as they may have distinct functions in tumorigenesis.

After establishing the necessity of STAT5b for maximal migration of breast cancer cells to serum, we investigated the mechanism by which this occurs. Interestingly, the migratory function of STAT5b does not require phosphorylation at tyrosine 699 (Y699) or the C-terminal transactivation domain (TAD), evidenced by equivalent rescue of migration with either wt-, Y699F-, or dn-STAT5b (Figure 2). Phosphorylation of Y699 is a hallmark of STAT5b transcriptional activation. If this residue is mutated to a phenylalanine, such that it cannot be phosphorylated (Y699F), the resulting STAT5b mutant is transcriptionally inactive [17,20]. Dn-STAT5b is also transcriptionally inactive because of the loss of the TAD and the ability to bind transcriptional cofactors [14]. Although STAT proteins are predominantly thought of as transcription factors that function in the nucleus, recent work identified a non-transcriptional, cytoplasmic role for unphosphorylated STAT3 in regulating tubulin dynamics [26]. Unphosphorylated STAT dimers take on an antiparallel configuration through interactions between the DNA-binding domains [27,28]. In this conformation, the SH2 domains are on opposite sides of the structure and are free to interact with phosphorylated tyrosines of other proteins. The necessity of the SH2 domain of STAT5b in migration is consistent with a cytoplasmic function of unphosphorylated STAT5b. We propose that STAT5b uses its SH2 domain to act as a scaffolding protein, bringing together signaling molecules necessary for efficient, directional migration. Although migration is inhibited to similar levels after STAT5b knockdown or blocking of β_1_-integrin (Figure 3), it remains to be determined whether STAT5b interacts directly with β_1_-integrin or if this effect is indirect. There are many tyrosine-phosphorylated proteins in the cytoplasm involved in migratory signaling with which STAT5b may interact, and future studies are aimed at uncovering these associations.

Loss of STAT5b leads to a polarity defect that impedes directional movement (Figure 4). During spreading on fibronectin, STAT5b-knockdown cells take on a remarkable phenotype distinguished by the formation of multiple, unstable protrusions (Figure 5). No defect is found in initial attachment, and once attached, protrusions extend rapidly. However, these protrusions are highly dynamic, and over time, they contract back into the cell. This phenotype is indicative of disrupted equilibrium between Rho family GTPases. Rac is localized predominantly at the leading edge, with Rho in the tail. Normal ratios of Rho and Rac lead to Rac-mediated formation and spreading of broad lamellipodia in the front of the cell, followed by Rho-mediated tail retraction, overall resulting in directional migration toward a stimulus [29]. Increased Rho activity in the front of the cell would disrupt this equilibrium and could account for the multiple protrusions and increased contraction at the front of the cell seen with STAT5b knockdown. Based on the data presented here, we postulate that unphosphorylated STAT5b mediates migration of breast cancer cells through regulation of cytoplasmic Rho GTPase family signaling.

In summary, these studies are the first to report a role for STAT5b in the migration of breast cancer cells. It is well established that STAT5b positively regulates breast cancer cell proliferation and survival, two processes important for initial tumor formation and growth. These data implicate STAT5b in the later stages of tumorigenesis also, such as migration. Future studies will further elucidate the mechanism by which STAT5b exerts its effect on migration, thereby broadening our understanding of how STAT5b promotes tumorigenesis and possibly metastasis. This will facilitate the long-term goal of defining conditions whereby STAT5b would be an effective therapeutic target for the treatment of breast cancer.

## Conclusions

Our studies demonstrate a novel function of STAT5b in breast cancer cell migration. The importance of STAT5b in mediating breast cancer cell proliferation and survival has already been established. This work is the first to show a positive regulatory role for STAT5b in breast cancer cell migration. Therefore, STAT5b not only may function in the initiation of tumorigenesis, through its pro-proliferative and pro-survival signaling, but also may promote tumor progression by mediating migration.

## Abbreviations

BSA: bovine serum albumin; DMEM: Dulbecco's modified eagle's medium; DMSO: dimethyl sulfoxide; dn: dominant-negative; EGF: epidermal growth factor; EGFR: epidermal growth factor receptor; F: phenylalanine; FBS: fetal bovine serum; FN: fibronectin; GFP: green fluorescent protein; GH: growth hormone; HA: hemagglutinin; K: lysine; LPA: lysophosphatidic acid; PBS: phosphate-buffered saline; Prl: prolactin; R: arginine; RFP: red fluorescent protein; SCCHN: squamous cell carcinoma of the head and neck; SH2: Src homology domain 2; STAT: signal transducer and activator of transcription; TAD: transactivation domain; TIRF: total internal reflection fluorescence; VN: vitronectin; wt: wild type; Y: tyrosine.

## Competing interests

The authors declare that they have no competing interests.

## Authors' contributions

TMB, CMS, and JTP designed the studies, and TMB performed the experiments. JZ assisted with the TIRF microscopy. All authors analyzed the data, and TMB drafted and revised the manuscript. All authors read and approved the final manuscript.

## References

[B1] Darnell JE (2002). Transcription factors as targets for cancer therapy. Nat Rev Cancer.

[B2] Bowman T, Garcia R, Turkson J, Jove R (2000). STATs in oncogenesis. Oncogene.

[B3] Turkson J, Jove R (2000). STAT proteins: novel molecular targets for cancer drug discovery. Oncogene.

[B4] Matsumura I, Kitamura T, Wakao H, Tanaka H, Hashimoto K, Albanese C, Downward J, Pestell RG, Kanakura Y (1999). Transcriptional regulation of the cyclin D1 promoter by STAT5: its involvement in cytokine-dependent growth of hematopoietic cells. EMBO J.

[B5] de Groot RP, Raaijmakers JA, Lammers JW, Koenderman L (2000). STAT5-dependent cyclin D1 and Bcl-xL expression in Bcr-Abl-transformed cells. Mol Cell Biol Res Commun.

[B6] Silva M, Benito A, Sanz C, Prosper F, Ekhterae D, Nunez G, Fernandez-Luna JL (1999). Erythropoietin can induce the expression of bcl-x(L) through Stat5 in erythropoietin-dependent progenitor cell lines. J Biol Chem.

[B7] Horita M, Andreu EJ, Benito A, Arbona C, Sanz C, Benet I, Prosper F, Fernandez-Luna JL (2000). Blockade of the Bcr-Abl kinase activity induces apoptosis of chronic myelogenous leukemia cells by suppressing signal transducer and activator of transcription 5-dependent expression of Bcl-xL. J Exp Med.

[B8] Nieborowska-Skorska M, Hoser G, Kossev P, Wasik MA, Skorski T (2002). Complementary functions of the antiapoptotic protein A1 and serine/threonine kinase pim-1 in the BCR/ABL-mediated leukemogenesis. Blood.

[B9] Kazansky AV, Spencer DM, Greenberg NM (2003). Activation of signal transducer and activator of transcription 5 is required for progression of autochthonous prostate cancer: evidence from the transgenic adenocarcinoma of the mouse prostate system. Cancer Res.

[B10] Sultan AS, Xie J, LeBaron MJ, Ealley EL, Nevalainen MT, Rui H (2005). Stat5 promotes homotypic adhesion and inhibits invasive characteristics of human breast cancer cells. Oncogene.

[B11] Liu X, Robinson GW, Wagner KU, Garrett L, Wynshaw-Boris A, Hennighausen L (1997). Stat5a is mandatory for adult mammary gland development and lactogenesis. Genes Dev.

[B12] Udy GB, Towers RP, Snell RG, Wilkins RJ, Park SH, Ram PA, Waxman DJ, Davey HW (1997). Requirement of STAT5b for sexual dimorphism of body growth rates and liver gene expression. Proc Natl Acad Sci USA.

[B13] Kloth MT, Catling AD, Silva CM (2002). Novel activation of STAT5b in response to epidermal growth factor. J Biol Chem.

[B14] Kloth MT, Laughlin KK, Biscardi JS, Boerner JL, Parsons SJ, Silva CM (2003). STAT5b, a mediator of synergism between c-Src and the epidermal growth factor receptor. J Biol Chem.

[B15] Riggins RB, Thomas KS, Ta HQ, Wen J, Davis RJ, Schuh NR, Donelan SS, Owen KA, Gibson MA, Shupnik MA, Silva CM, Parsons SJ, Clarke R, Bouton AH (2006). Physical and functional interactions between Cas and c-Src induce tamoxifen resistance of breast cancer cells through pathways involving epidermal growth factor receptor and signal transducer and activator of transcription 5b. Cancer Res.

[B16] Weaver AM, Silva CM (2006). Modulation of signal transducer and activator of transcription 5b activity in breast cancer cells by mutation of tyrosines within the transactivation domain. Mol Endocrinol.

[B17] Fox EM, Bernaciak TM, Wen J, Weaver AM, Shupnik MA, Silva CM (2008). Signal transducer and activator of transcription 5b, c-Src, and epidermal growth factor receptor signaling play integral roles in estrogen-stimulated proliferation of estrogen receptor-positive breast cancer cells. Mol Endocrinol.

[B18] Yamashita H, Iwase H, Toyama T, Fujii Y (2003). Naturally occurring dominant-negative Stat5 suppresses transcriptional activity of estrogen receptors and induces apoptosis in T47D breast cancer cells. Oncogene.

[B19] Yamashita H, Nishio M, Fujii Y, Iwase H (2004). Dominant-negative stat5 inhibits growth and induces apoptosis in T47D-derived tumors in nude mice. Cancer Sci.

[B20] Weaver AM, Silva CM (2007). Signal transducer and activator of transcription 5b: a new target of breast tumor kinase/protein tyrosine kinase 6. Breast Cancer Res.

[B21] Li X, Leung S, Kerr IM, Stark GR (1997). Functional subdomains of STAT2 required for preassociation with the alpha interferon receptor and for signaling. Mol Cell Biol.

[B22] Muller A, Homey B, Soto H, Ge N, Catron D, Buchanan ME, McClanahan T, Murphy E, Yuan W, Wagner SN, Barrera JL, Mohar A, Verategui E, Zlotnik A (2001). Involvement of chemokine receptors in breast cancer metastasis. Nature.

[B23] Hayman EG, Ruoslahti E (1979). Distribution of fetal bovine serum fibronectin and endogenous rat fibronectin in extracellular matrix. J Cell Biol.

[B24] Xi S, Zhang Q, Gooding WE, Smithgall TE, Grandis JR (2003). Constitutive activation of Stat5b contributes to carcinogenesis in vivo. Cancer Res.

[B25] Kazansky AV, Rosen JM (2001). Signal transducers and activators of transcription 5B potentiates v-Src-mediated transformation of NIH-3T3 cells. Cell Growth Differ.

[B26] Ng DC, Lin BH, Lim CP, Huang G, Zhang T, Poli V, Cao X (2006). Stat3 regulates microtubules by antagonizing the depolymerization activity of stathmin. J Cell Biol.

[B27] Mao X, Ren Z, Parker GN, Sondermann H, Pastorello MA, Wang W, McMurray JS, Demeler B, Darnell JEJ, Chen X (2005). Structural bases of unphosphorylated STAT1 association and receptor binding. Mol Cell.

[B28] Neculai D, Neculai AM, Verrier S, Straub K, Klumpp K, Pfitzner E, Becker S (2005). Structure of the unphosphorylated STAT5a dimer. J Biol Chem.

[B29] Burridge K, Doughman R (2006). Front and back by Rho and Rac. Nature Cell Biol.

